# Der schwer verletzte ältere Fahrradfahrer – Auswertung des TraumaRegister DGU®: Retrospektive, multizentrische Querschnittstudie anhand des TraumaRegister DGU®

**DOI:** 10.1007/s00113-022-01286-6

**Published:** 2023-03-29

**Authors:** Konrad Fuchs, Roman Backhaus, Martin C. Jordan, Rolf Lefering, Rainer H. Meffert, Fabian Gilbert, das TraumaRegister DGU

**Affiliations:** 1 Klinik und Poliklinik für Unfall‑, Hand‑, Plastische und Wiederherstellungschirurgie, Julius-Maximilians-Universität Würzburg, Würzburg, Deutschland; 2 MUM – Muskuloskelettales Universitätszentrum München, Ludwigs-Maximilians-Universität München, Campus Innenstadt, Ziemssenstr. 5, 80336 München, Deutschland; 3 Sektion Notfall‑, Intensivmedizin und Schwerverletztenversorgung (Sektion NIS), Deutsche Gesellschaft für Unfallchirurgie (DGU), Berlin, Deutschland

**Keywords:** Polytrauma, Alterstraumatologie, Kopfverletzung, Notfallversorgung, Registerstudie, Polytrauma, Geriatric trauma, Head injury, Emergency care, Registry study

## Abstract

**Hintergrund:**

Entgegen dem Trend sinkender Verkehrstoter nimmt die Zahl der getöteten Fahrradfahrer in Deutschland in den letzten Jahren kontinuierlich zu. Mit zunehmender Popularität des Fahrradfahrens in allen Altersklassen erhöht sich die Anzahl an Unfällen mit z. T. schweren Verletzungen. Im Zuge dessen stellt sich die Frage, welchen Einfluss das Alter auf die Art und Schwere der Verletzungen, die Überlebenswahrscheinlichkeit und die Krankenhausverweildauer bei schwer verletzten Fahrradfahrern hat.

**Methoden:**

Es wurde eine retrospektive Auswertung der Daten des TraumaRegister DGU® (TR-DGU) der Jahre 2010–2019 durchgeführt. Alle schwer verletzten Fahrradfahrer mit einem maximalem MAIS von 3 (Abbreviated Injury Scale) von 3+ (*n* = 14.651) im TR-DGU wurden in diese Studie eingeschlossen und die vorliegenden Parameter ausgewertet. Es erfolgte eine Unterteilung in 3 Altersgruppen (60 bis 69, 70 bis 79 und ≥ 80 Jahre) und eine Kontrollgruppe (20 bis 59 Jahre).

**Ergebnisse:**

Verletzungen des Schädels traten mit 64,2 % mit Abstand am häufigsten auf. Es zeigte sich eine deutliche Zunahme der schweren Kopfverletzungen in der Gruppe der über 60-Jährigen. Mit steigendem Alter nahmen des Weiteren die Wahrscheinlichkeit einer präklinischen Intubation, die Katecholaminpflichtigkeit, die Intensiv- und Krankenhausverweildauer sowie die Sterblichkeit zu.

**Schlussfolgerung:**

Kopfverletzungen stellen die häufigste schwere Verletzung, insbesondere bei älteren Fahrradfahrern, dar. Da das Helmtragen im TraumaRegister DGU® im Auswertungszeitraum nicht erfasst wurde, kann auf dessen Effekt kein Rückschluss gezogen werden. Ein höheres Alter korreliert des Weiteren mit einer längeren Krankenhausverweildauer und einer höheren Sterblichkeit, stellt jedoch keinen unabhängigen Risikofaktor zum Versterben bei einem schwer verletzten Patienten dar.

**Graphic abstract:**

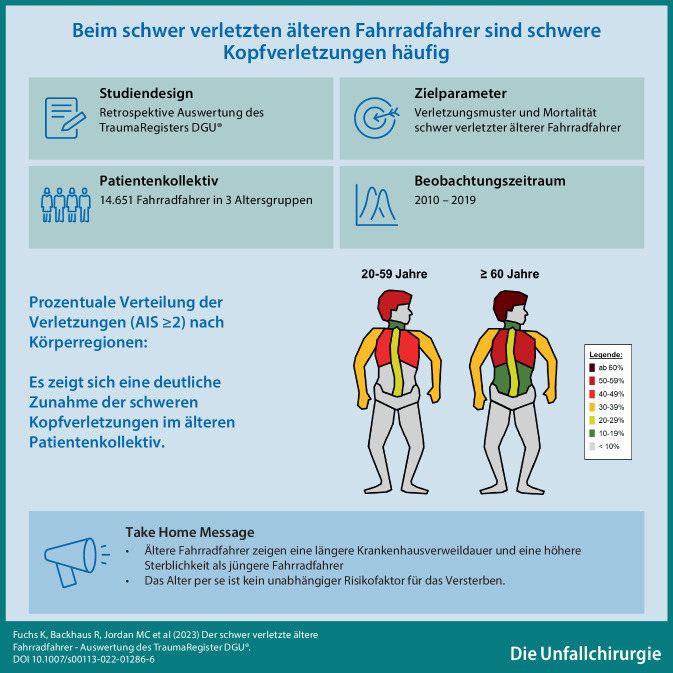

## Abstract (en)

**Background:**

Contrary to the trend of decreasing traffic fatalities, the number of cyclists killed in Germany has been steadily increasing in recent years. With the increasing popularity of cycling in all age groups, the number of accidents with sometimes serious injuries is rising. In the course of this, the question arises what influence age has on the type and severity of injuries, the probability of survival and the length of hospital stay in seriously injured cyclists.

**Methods:**

A retrospective analysis of data from the TraumaRegister DGU® (TR-DGU) from 2010 to 2019 was performed. All severely injured cyclists with a maximum abbreviated injury scale (MAIS) of 3+ (*n* = 14,651) in the TR-DGU were included in this study and the available parameters were evaluated. A subdivision into three age groups (60–69, 70–79, and ≥ 80 years) and a control group (20–59 years) was carried out.

**Results:**

Injuries to the head were by far the most common, accounting for 64.2%. There was a marked increase in severe head injuries in the 60-plus years age group. Furthermore, with increasing age, the probability of prehospital intubation, catecholamine requirement, intensive care and hospital length of stay, and mortality increased.

**Conclusion:**

Head injuries represent the most common serious injury, especially among older cyclists. As helmet wearing was not recorded in the TraumaRegister DGU® during the evaluation period, no conclusion can be drawn about its effect. Furthermore, a higher age correlates with a longer hospital stay and a higher mortality, but does not represent an independent risk factor for death in severely injured patients.

## Einleitung

Die Anzahl der Verkehrstoten ist seit Anfang der 1970er-Jahre in Deutschland deutlich rückläufig [20]. Die Gründe hierfür sind zahlreich. Sie reichen von der Einführung einer Promillegrenze, der Gurtpflicht bis hin zur Helmpflicht bei Motorradfahrern. Dies betrifft jedoch v. a. die motorisierten Verkehrsteilnehmer. Entgegen der sinkenden Zahl der Verkehrstoten insgesamt, stieg laut Statistischem Bundesamt die Zahl der getöteten Fahrradfahrer von 2010 bis 2019 um 16,8 % [21]. Von den 445 Fahrradfahrern, die 2019 verstarben, waren 53,8 % 65 Jahre oder älter [21]. Verglichen mit den 2 anderen großen Verkehrsteilnehmergruppen, Pkw und Krafträder mit amtlichen Kennzeichen (KMK), stehen die Fahrradfahrer auf dem zweiten Platz bezüglich der Schwerverletzten im Straßenverkehr 2019 (Pkw 28.302, Fahrrad 15.176, KMK 9128) [1].

Die Daten des Statistischen Bundesamtes und die klinische Erfahrung deuten auf eine Zunahme an Fahrradunfällen mit schwerem Verletzungsmuster hin. Ferner steigt die Anzahl an älteren Personen, die mit dem Fahrrad verunfallen. Dieses besonders vulnerable Patientenkollektiv stellt die behandelnden Ärzte und Pflegekräfte vor besondere Herausforderungen. Eden et al. konnten bereits zeigen, dass schwer verletzte Motorradfahrer im Alter über 65 Jahren eine höhere Sterblichkeit, eine verlängerte Beatmungsdauer und eine längere Krankenhausverweildauer aufwiesen [3].

Aus diesen Gründen ist es erforderlich, dass die Daten zu schwer verletzten Fahrradfahrern der letzten 10 Jahre systematisch aufgearbeitet werden.

Es stellt sich ferner die Frage, wie sich das Alter auf die Art und Schwere der Verletzungen, die Überlebenswahrscheinlichkeit und die Krankenhausverweildauer bei schwer verletzten Fahrradfahrern auswirkt. Die Ausgangshypothese dieser Studie ist, dass ältere Patienten ein größeres Risikoprofil mitbringen und hierdurch ein schlechterer Outcome zu erwarten ist.

## Material und Methoden

Das Traumaregister der Deutschen Gesellschaft für Unfallchirurgie (TR-DGU) wurde 1993 gegründet. Ziel dieser multizentrischen Datenbank ist eine pseudonymisierte und standardisierte Dokumentation von Schwerverletzten. Die Daten werden prospektiv in 4 aufeinanderfolgenden Phasen gesammelt: A) präklinische Phase, B) Schockraum und anschließende OP-Phase, C) Intensivstation und D) Entlassung. Die Dokumentation beinhaltet detaillierte Informationen über Demografie, Verletzungsmuster, Komorbiditäten, präklinisches und klinisches Management, intensivmedizinischen Verlauf, wichtige Laborbefunde, einschließlich Transfusionsdaten, sowie das Outcome. Das Einschlusskriterium ist die Aufnahme in das Krankenhaus über den Schockraum mit anschließender Intensiv- oder Intermediate-Care-Überwachung oder Ankunft in der Klinik mit Vitalzeichen und Versterben vor Aufnahme auf die Intensivstation. Die Infrastruktur für Dokumentation, Datenmanagement und Datenanalyse wird von der Akademie der Unfallchirurgie GmbH (AUC), welche der Deutschen Gesellschaft für Unfallchirurgue (DGU) angegliedert ist, bereitgestellt. Die wissenschaftliche Führung liegt bei der Sektion Notfall‑, Intensivmedizin und Schwerverletztenversorgung der DGU (Sektion NIS). Über eine webbasierte Anwendung geben die teilnehmenden Kliniken ihre Daten pseudonymisiert in eine zentrale Datenbank ein. Wissenschaftliche Auswertungen werden nach einem in der Publikationsrichtlinie des TR-DGU festgeschriebenen Peer-Review-Verfahren genehmigt. Die teilnehmenden Kliniken sind primär in Deutschland (90 %) lokalisiert, aber eine zunehmende Anzahl von Kliniken aus anderen Ländern trägt ebenfalls Daten bei (zurzeit aus Österreich, Belgien, China, Finnland, Luxemburg, Slowenien, Schweiz, den Niederlanden und den Vereinigten Arabischen Emiraten). Derzeit fließen jährlich knapp 30.000 Fälle von über 650 Kliniken in die Datenbank ein. Die Beteiligung am TR-DGU ist freiwillig; für die dem TraumaNetzwerk DGU® zugehörigen Kliniken ist die Eingabe zumindest eines Basisdatensatzes zur Qualitätssicherung verpflichtend.

In dieser retrospektiven multizentrischen Querschnittstudie wurden Daten des TR-DGU zu allen Fahrradunfallopfern vom 01.01.2010 bis 31.12.2019 ausgewertet. Die Arbeit steht in Übereinstimmung mit der Publikationsrichtlinie des TR-DGU und ist registriert unter der TR-DGU-Projekt-ID 2020-013.

Die Behandlungsverläufe wurden in 4 Phasen aufgeteilt (präklinische Phase, Schockraum- und ggf. OP-Phase, intensivmedizinische Phase und Entlassung). Das Patientenkollektiv wurde ferner in 3 Altersgruppen: 60 bis 69, 70 bis 79 und ≥ 80 Jahre und eine Kontrollgruppe 20 bis 59 Jahre aufgeteilt.

Die Verletzungsschwere kann anhand des Injury Severity Score (ISS) berechnet werden. Der ISS kann einen Wert von 1 bis 75 annehmen und ergibt sich aus der Summe der Quadratur der Werte der maximalen Abreviated Injury Scale (MAIS) der 3 am schwersten verletzten Körperregionen. Die Mortalitätswahrscheinlichkeit von Schwerverletzten kann anhand des Revised Injury Severity Score II (RISC II) abgeschätzt werden. Er wurde anhand der Daten des TR-DGU entwickelt und beinhaltet die AIS (Abbreviated Injury Scale: Jede Verletzung wird mit einer Verletzungsschwere von 0 (keine Verletzung) bis 6 (tödliche Verletzung) bewertet) der 2 am schwersten verletzten Körperregionen, die Schwere einer möglichen Kopfverletzung (MAIS-Wert), das Alter, Geschlecht, die Pupillenreaktion und -größe, den ASA-Status vor dem Unfallereignis, die motorische Antwort (motorischer Wert der GCS), den Unfallmechanismus (stumpf/penetrierend), den systolischen Blutdruck, den Base Excess, die INR, den Hb und, ob präklinisch eine externe kardiopulmonale Reanimation durchgeführt wurde [10].

Es wurden alle verletzten Fahrradfahrer inkludiert, bei denen ein MAIS ≥ 3 festgestellt wurde. Es erfolgte eine Auswertung der in Tab. 1 genannten Daten, anhand derer zusätzlich der RISC II bestimmt wurde.

**Table 1:** 

Variable	20 bis 59 Jahre (*n* = 7895)	60 bis 69 Jahre (*n* = 2660)	70 bis 79 Jahre (*n* = 2830)	≥ 80 Jahre (*n* = 1246)
Geschlecht	72,9 % (*n* = 5754) ♂	70,9 % (*n* = 1885) ♂	67,8 % (*n* = 1919) ♂	70,3 % (*n* = 876) ♂
ASA-Klassifikation (ASA 3–4)	3,5 %	12,9 %	25,0 %	35,3 %
Katecholaminpflichtigkeit	6,6 %	9,7 %	10,1 %	15,2 %
GCS, präklinisch (% < 9)	19,3 %	23,4 %	26,5 %	26,5 %
Präklinische Intubation	23,0 %	23,6 %	28,8 %	30,2 %
Unfall am Wochenende (Freitag bis Sonntag)	46,8 %	40,3 %	39,2 %	35,9 %
Unfallzeitpunkt (Tag/Nacht)	59,1 %/40,9 %	68,2 %/31,8 %	76,6 %/23,4	79,6 %/20,4 %
Mortalität	4,7 %	8,7 %	15,2 %	28,8 %
ISS (Punkte)	19,8 (± 10)	20,7 (± 10,5)	22,4 (± 11,9)	23,6 (± 11,6)
RISC-II-Score	5,40 %	9,50 %	17,60 %	31,20 %
EK-Gabe im Schockraum	5,3 %	5,3 %	7,3 %	9,0 %
Mortalitätsanalyse nach SMR	0,89 [0,798; 0,981]	0,942 [0,823; 1,031]	0,902 [0,822; 0,983]	0,941 [0,856; 1,026]
Antikoagulative Medikation bzw. Thrombozytenaggregationshemmer in Dauermedikation	1,4 %	7,0 %	15,4 %	20,6 %
Koagulopathie (Quick-Wert ≤ 60 %, PTT ≥ 40, INR ≥ 1,4)	5,4 %	7,7 %	14,7 %	20,6 %
Intubationsdauer (Tage)^a^	8 (SD 9,9)	9,4 (± 11,3)	10 (± 11,6)	8,6 (± 11,3)
Liegedauer, Intensivstation (Tage) Überlebende	5,7 (SD 9)	6,9 (± 10,4)	8,3 (± 11,7)	8,1 (± 11,2)
Liegedauer, Intensivstation (Tage) Verstorbene	4,7 (SD 6,2)	6,3 (± 10,6)	6,9 (± 11,7)	6,1 (± 11)
Liegedauer, Krankenhaus (Tage) Überlebende	15,2 (SD 16,4)	17 (± 14,7)	18,8 (± 16,7)	19,3 (± 16)
Liegedauer, Krankenhaus (Tage) Verstorbene	5,5 (SD 7)	7,5 (± 11,9)	8,6 (± 16,2)	7,5 (± 13,2)

*SD* Standardabweichung, *GCS* Glasgow Coma Scale, *ISS* Injury Severity Score

^a^Der hier aufgeführte Wert bezieht sich nicht auf alle verunfallten Fahrradfahrer im TR-DGU, sondern nur auf alle tatsächlich intubierten Patienten

Das standardisierte Mortalitätsverhältnis (SMR) wurde anhand des Quotienten aus erwarteter und gemessener Mortalität berechnet. Ein Wert > 1 bedeutet, dass mehr Personen in dem vorhandenen Kollektiv verstarben als erwartet.

## Statistische Auswertung

Die erhobenen Daten wurden mittels SPSS Version 24 (IBM Inc, Armonk, NY, USA) ausgewertet. Es erfolgten eine quantitative Bestimmung (*n*) und die Berechnungen der prozentualen Verteilungen. Bei metrischen Daten wurde ferner der Mittelwert und die Standardabweichung (SD) errechnet.

## Ergebnisse

**Figure 1:**
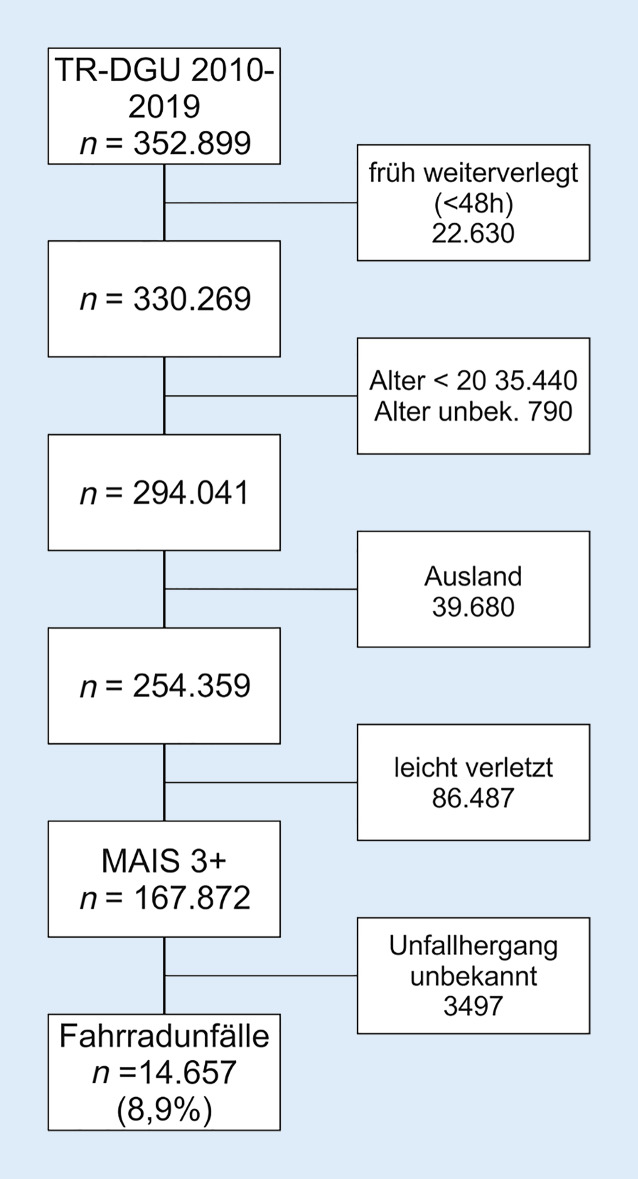

**Figure 2:**
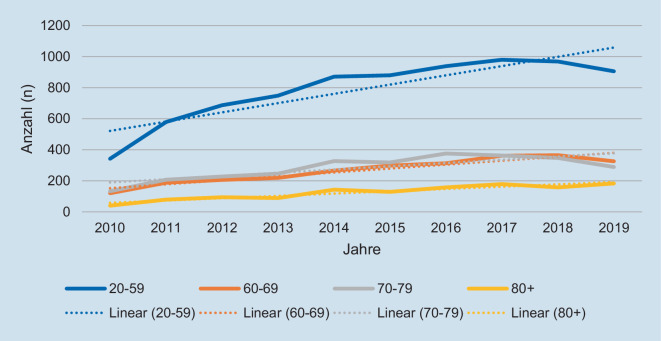

**Figure 3:**
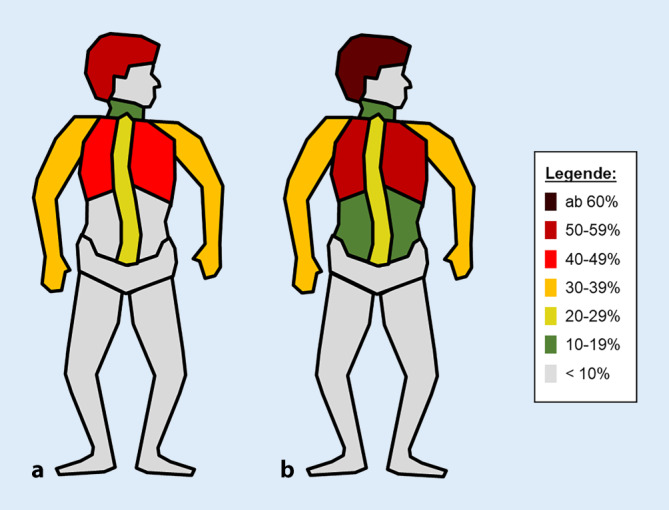

Im Zeitraum vom 01.01.2010 bis 31.12.2019 wurden 14.657 schwer verletzte Fahrradfahrer mit einem Alter ≥ 20 Jahren im TR-DGU erfasst (Abb. 1). Betrachtet man die prozentuale Verteilung bei der Unfallart, aufgeteilt in 2 Altersgruppen (20 bis 59 und ≥ 60 Jahre), zeigt sich eine stetige Zunahme in beiden Altersgruppen im beobachteten Zeitraum (Abb. 2). Verletzungen wurden ab einem AIS ≥ 2 festgehalten. Insgesamt zeigte sich eine ähnliche Verteilung der Verletzungen zwischen beiden Altersgruppen (Abb. 3). In der älteren Gruppe konnte ein deutlich höherer Anteil an schweren Kopfverletzungen (Alter 20 bis 59 Jahre: 59,7 %; ≥ 60 Jahre: 69,3 %) festgestellt werden. Schwere Verletzungen des Thorax traten in beiden Alterskollektiven am zweithäufigsten auf (Alter 20 bis 59 Jahre 48,8 %; ≥ 60 50 %). Danach folgten schwere Verletzungen der Arme (Alter 20 bis 59 35,5 %; ≥ 60 33,2 %), der Wirbelsäule (Alter 20 bis 59 Jahre 22,8 %; ≥ 60 21,5 %), der Beine (Alter 20 bis 59 15,8 %; ≥ 60 19,2 %) des Beckens (Alter 20 bis 59 10,1 %; ≥ 60 14,9 %) und des Abdomens (Alter 20 bis 59 11,1 %; ≥ 60 8,3 %).

**Figure 4:**
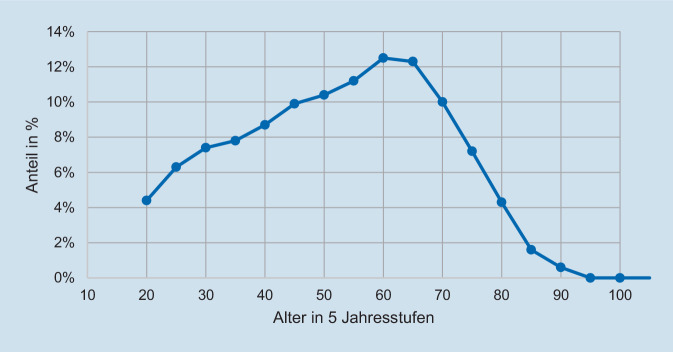

Werden die Altersgruppen in 10-Jahres-Abschnitte aufgeschlüsselt, ist, prozentual gesehen, bei der Gruppe der 60- bis 69-Jährigen der größte Anteil an schwer verletzten Fahrradfahrern (Abb. 4) zu finden.

**Figure 5:**
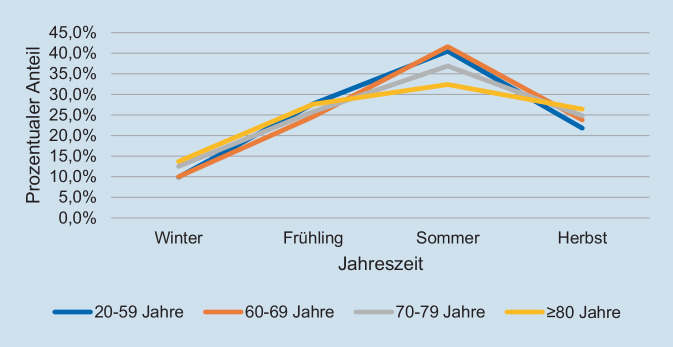

Betrachtet man die Verteilung der Altersgruppen nach Jahreszeit, fällt eine Häufung der Gruppe der 20- bis 59-Jährigen im Sommer auf, während in den älteren Gruppen eine gleichmäßigere Verteilung über das Jahr festzustellen ist (Abb. 5).

Insgesamt kann in dem untersuchten Patientenkollektiv unter allen Altersgruppen eine deutliche Mehrheit an männlichen Verletzten festgestellt werden (Tab. 1). Ein erhöhtes Alter geht mit einem höheren ASA-Score einher (Tab. 1). Ferner steigt mit zunehmendem Alter die Schwere der Verletzungen, gemessen durch den ISS, an (Tab. 1). Mit höherem Alter steigen die Katecholaminbedürftigkeit, die Wahrscheinlichkeit einer präklinischen Intubation, der intensivmedizinische und stationäre Aufenthalt insgesamt, die Mortalität und der RISC-II-Score. Im jüngeren Patientenkollektiv kann hingegen häufiger ein Alkoholeinfluss festgestellt werden (Tab. 2).

**Table 2:** 

BAK Wert	Anteil 20–59 Jahre (%)	Anteil 60–69 Jahre (%)	Anteil 70–79 Jahre (%)	Anteil > 80 Jahre (%)	Gesamt (%)
**<** **0,01** **‰**	82,5	88,4	96	97,5	87,5
**0,5–2** **‰**	11,2	8,6	3,7	2,5	8,5
**>** **2** **‰**	6,3	3	0,3	0	4

## Diskussion

Fahrradfahren spielt eine wichtige Rolle als Freizeitaktivität und umweltfreundliches Fortbewegungsmittel. Im Straßenverkehr sind Fahrradfahrer jedoch aufgrund ihrer Exponiertheit bei Unfällen gefährdet. Bisherige klinische Studien untersuchen häufig die Rolle des Fahrradhelms auf die Reduktion der Mortalität bei Fahrradunfällen [11, 13, 16] oder auf Unfallursachen und beziehen sich in vielen Fällen auf das nichteuropäische Ausland.

Bezüglich der Schwerverletztenversorgung bei Fahrradunfällen ist die Studienlage in Deutschland begrenzt. Juhra et al. führten von 2009–2010 eine Studie zu Fahrradunfällen in Münster durch [9]. In dem Patientenkollektiv von 2250 Verunfallten waren die oberen (36,8 %) und unteren (29,9 %) Extremitäten am häufigsten verletzt, gefolgt vom Schädel (25,7 %). Es handelte sich bei dem Patientenkollektiv jedoch nicht lediglich um schwer verletzte Fahrradfahrer, sondern um alle erfassten Fahrradunfälle (durch Kliniken oder durch die Polizei). Strohm et al. werteten von 2003 bis 2004 23 polytraumatisierte Fahrradfahrer in Freiburg aus [24]. Hier zeigte sich das Schädel-Hirn-Trauma als führende Verletzung. Die genannten Studien bezogen sich auf eine Stadt oder begrenzte Region. Regionale Untersuchungen können jedoch kein ganzheitliches Bild der Schwerverletztenversorgung von Fahrradfahrern darstellen. In einer belgischen Studie konnten deutliche regionale Unterschiede bezüglich der Unfallhäufigkeit und der Mortalität festgestellt werden [25]. Ein hohes Aufkommen an Fahrradfahrern ging mit einem geringeren Risiko einher, beim Fahrradfahren schwer verletzt oder getötet zu werden [25]. Eine bessere Fahrradinfrastruktur führt hingegen zu einer Senkung der Unfallwahrscheinlichkeit [15, 23, 26].

Zwipp et al. werteten die GIDAS-Datenbank („German In-Depth Accident Study“) zwischen 1999 und 2008 aus [28]. In dieser Studie wurden alle verletzten Fahrradfahrer ausgewertet, ungeachtet der Verletzungsschwere. Bei den 6718 verletzten Fahrradfahrern waren die Beine (56,9 %) am häufigsten betroffen, gefolgt vom Kopf (44,2 %) und den Armen (44,0 %). Helfen et al. werteten die Daten des TR-DGU im Zeitraum von 2002 bis 2010 (*n* = 2817) bezüglich schwer verletzter Fahrradfahrer aus [6]. In dieser Auswertung des TR-DGU konnte bereits festgestellt werden, dass Kinder und Senioren zur Hochrisikogruppe gehören. Seit Erhebung der Daten haben die Verkaufszahlen von Fahrrädern und E‑Bikes drastisch zugenommen, und es kam zu einer Vielzahl von Verkehrsprojekten, um die Sicherheit von Fahrradfahrern zu erhöhen [12]. Ferner ist die Zahl der am TR-DGU teilnehmenden Kliniken deutlich gestiegen. Es ist daher erforderlich, eine aktuelle und umfangreiche Analyse der Daten zu schwer verletzten Fahrradfahrern in Deutschland durchzuführen.

Insgesamt belegen die hier vorliegenden Daten eine Zunahme des Anteils an schwer verletzten Fahrradfahrern in allen Altersgruppen. Da Fahrräder im Gegensatz zu Pkw und KMK nicht zugelassen werden müssen, kann das Statistische Bundesamt die Anzahl der im Verkehr teilnehmenden Fahrräder nicht erfassen. Die Pressemitteilungen des Zweirad-Industrie-Verbandes legen jedoch eine Zunahme der Verkaufszahlen von Fahrrädern (4,31 Mio. in 2019) in Deutschland nahe [27]. Da keine verlässlichen Daten zur Fahrradnutzung in Deutschland existieren, bleibt nicht endgültig geklärt, ob die Zunahme des Anteils an schwer verletzten Fahrradfahrern durch eine höhere Gefährdung im Straßenverkehr oder eine generelle Zunahme an Fahrradfahrern begründet ist.

In der vorliegenden Studie kann gezeigt werden, dass schwere Verletzungen (AIS ≥ 2) des Kopfes im TR-DGU am häufigsten auftraten. Insbesondere ältere Verkehrsteilnehmer sind hiervon betroffen (Alter 20 bis 59 Jahre: 59,7 %; ≥ 60: 69,3 %). Es sollte daher bei der Triage von Fahrradfahrern insbesondere auf Kopfverletzungen geachtet werden. Aktuell wird im TR-DGU nicht standardisiert erfasst, ob die Verletzten einen Helm trugen. Es kann daher nicht berücksichtigt werden, ob ältere Schwerverletzte seltener einen Helm trugen, oder ob andere Ursachen hierfür infrage kommen. Zwipp et al. beschreiben bei Patienten über 70 Jahren eine Fahrradhelmtragefrequenz von lediglich 3 % [28]. In Deutschland wird die Tragefrequenz von Fahrradhelmen über alle Altersgruppen zwischen 4,1 und 14 % beziffert [14, 28, 29]. Mit steigendem Alter nimmt das Reaktionsvermögen schrittweise ab [5], und es wäre prinzipiell denkbar, dass es hierdurch bei einem Sturz häufiger zu einem Kopfanprall kommt. Vor diesem Hintergrund wäre die Erfassung im TR-DGU, ob ein Fahrradhelm beim Unfall getragen wurde, sinnvoll. Eine Vielzahl an Studien zeigt eine deutliche Reduktion der Mortalität durch das Tragen von Fahrradhelmen [2, 7, 11, 13]. Anhand der hier ausgewerteten Daten sollten daher insbesondere ältere Verkehrsteilnehmer einen Fahrradhelm tragen, da sie aktuell besonders häufig schwere Kopfverletzungen erleiden.

Bezüglich des weiteren Verletzungsmusters zeigt sich bei dem älteren Patientenkollektiv eine Vermehrung von Verletzungen des Beckens, bei sonst sehr ähnlichen prozentualen Verteilungen der restlichen Körperregionen. In der Literatur wurde dies bisher noch nicht beschrieben. Mögliche Ursache hierfür könnte eine höhere Osteoporoserate in der älteren Bevölkerungsgruppe sein [8].

In der Gruppe der 20 -bis 59-Jährigen kann ein deutlicher Anstieg an Schwerverletzten während der Sommermonate festgestellt werden. Es ist zu vermuten, dass dies durch eine Zunahme der Fahrradpendler, aber auch durch sportliche Aktivitäten, wie Rennrad- oder Mountainbikefahren, verursacht wird. Im Kollektiv der älteren Patienten ist dieser Anstieg hingegen weniger ausgeprägt. Es ist daher zu vermuten, dass insbesondere ältere Personen unabhängig von der Jahreszeit Fahrradfahren. Auffällig ist ferner, dass jüngere Fahrer, verglichen mit dem älteren Patientenkollektiv, wesentlich häufiger in der Nacht verunfallen. Ob dies durch mehr Pendlerfahrten oder im Rahmen der Freizeit geschieht, lässt sich nicht klären.

Alkoholeinfluss spielt insbesondere im jüngeren Patientenkollektiv eine Rolle. Auch BAK-Werte über 1,6‰ wurden festgestellt. Ab diesem Wert besteht nach §316 StGB eine absolute Fahruntüchtigkeit für Fahrradfahrer. Mit über 17 % der schwer verletzten Fahrradfahrer unter Alkoholeinfluss der 20- bis 59-Jährigen sollte hier vermehrt Aufklärungsarbeit bezüglich der Risiken erfolgen.

Die vorliegenden Daten zeigen bezüglich der Sterblichkeit eine Zunahme mit dem Alter. Bei dem Patientenkollektiv ≥ 80 Jahre lag die Mortalität sogar bei 28,8 %. Mögliche Gründe hierfür sind eine stetige Zunahme des durchschnittlichen ISS, die vermehrten schweren Verletzungen des Schädels und Beckens sowie die Zunahme des ASA-Scores mit steigendem Alter. Diese Gründe führen wahrscheinlich auch dazu, dass das ältere Patientenkollektiv häufiger katecholamin- und/oder intubationspflichtig wurden. Die zentrale Frage ist jedoch, ob Fahrradfahren, verglichen mit anderen schwer verletzten Patienten, zu einer höheren Sterblichkeit führt. Dies kann anhand des SMR ausgewertet werden. In allen Gruppen war der Wert < 1 (Tab. 1). Es kann somit gezeigt werden, dass das Alter beim Fahrradfahren keinen unabhängigen Risikofaktor zum Versterben bei einem schwer verletzten Patienten darstellt. Dementsprechend sollte Fahrradfahren in allen Bevölkerungsgruppen gefördert werden, da es zusätzlich einen positiven Einfluss auf die Gesundheit und Umwelt hat [4, 17, 18].

Die Anzahl an E‑Bikes nimmt stetig zu, v. a. bei der älteren Bevölkerung [22]. Ob ein Fahrrad eine elektronische Unterstützung besitzt, wird im TR-DGU erst seit 2020 erfasst und ist somit nicht auswertbar. Siman-Tov et al. konnten bei einer Auswertung des israelischen Traumaregisters bereits zeigen, dass E‑Bike-Fahrer schwerere Verletzungen des Kopfes erleiden, häufiger eine operative Intervention und einen längeren Krankenhausaufenthalt benötigen [19]. Welchen Einfluss der Elektromotor auf das Patientenkollektiv und Verletzungsmuster hat, sollte daher in zukünftigen Studien des TR-DGU geklärt werden.

## Methodische Stärken und Schwächen der Studie

Bei dieser Studie handelt es sich um eine multizentrische, retrospektive Querschnittstudie. Die Daten werden prospektiv-konsekutiv von den teilnehmenden Kliniken erfasst und an das TR-DGU übertragen. Durch eine zunehmende flächendeckende deutschlandweite Teilnahme der Kliniken lassen sich allgemeine Aussagen zur Schwerverletztenversorgung treffen.

Die erfassten Daten sind sehr umfangreich, beinhalten aber nicht für alle Fragestellungen die relevanten Informationen (z. B. die Fragen, ob ein Helm getragen wurde, oder ob es sich um einen E‑Bike-Unfall handelte). Ferner können Behandlungsverläufe anhand der vorliegenden Daten nur begrenzt nachvollzogen werden. Hier sind weiterhin monozentrische Datenerhebungen erforderlich.

Die Verkehrspolitik befindet sich aktuell in einem Wandel. Umweltfreundlichere Fortbewegungsmittel müssen in Zukunft in das bestehende Verkehrssystem integriert werden oder dieses teilweise ersetzen. Als kostengünstiges und gesundheitsförderndes Fortbewegungsmittel wird das Fahrrad auch in Zukunft eine zentrale Rolle im Straßenbild spielen.

Da der Anteil der schwer verletzten Fahrradfahrer in allen Altersgruppen zunimmt, sollten Konzepte entwickelt werden, um dieses, im Straßenverkehr besonders vulnerable Kollektiv besser vor und bei Unfällen zu schützen.

## Fazit für die Praxis

- Der Anteil der schwer verletzten Fahrradfahrer nimmt in allen Altersgruppen zu.
- Die Kopfverletzung ist die häufigste schwere Verletzung und betrifft insbesondere ältere Patienten. Es sollte daher insbesondere beim Triagieren auf diese geachtet werden.
- Ältere Fahrradfahrer sind bei Eintreffen im Schockraum häufiger im systemischen Schock, haben eine höhere Sterblichkeit und einen längeren Intensiv- und generellen Krankenhausaufenthalt.
- Fahrradfahren stellt jedoch keinen unabhängigen Risikofaktor, an einem Polytrauma zu versterben, dar.
- Weitere Maßnahmen zum Schutz von Fahrradfahrern sind erforderlich.
